# Potential role of breast MRI to identify patients with high-risk lesions who might avoid surgery: a systematic review and meta-analysis

**DOI:** 10.1007/s00330-025-12291-9

**Published:** 2026-01-17

**Authors:** Giulia Vatteroni, Nina Pirringer-Pötsch, Paola Clauser, Pascal A. T. Baltzer

**Affiliations:** 1 Department of Biomedical Sciences, Humanitas University, Milan, Italy; 2 Department of Biomedical Imaging and Image-guided Treatment, High field Magnetic Resonance Center (HFMRC), Medical University of Vienna, Vienna, Austria

**Keywords:** Breast cancer, Magnetic resonance imaging, B3 lesions, Lesions of uncertain malignant potential, Patient management

## Abstract

**Objective:**

This systematic review and meta-analysis investigate the added value of contrast-enhanced breast MRI (CE-MRI) to rule out malignancy in patients with high-risk (B3) lesions diagnosed at image-guided biopsy.

**Materials and methods:**

A systematic review and meta-analysis were performed using predefined criteria. Eligible English-language articles published until August 2024 focused on CE-MRI in high-risk lesions. Two reviewers extracted data on true positives (TP), false positives (FP), true negatives (TN), and false negatives (FN). Sensitivity, specificity, negative and positive likelihood ratios were calculated using a bivariate random-effects model. Fagan nomograms identified the maximum pretest probability at which post-test probabilities of a negative MRI matched the 2% malignancy threshold used for downgrading BI-RADS 4 to 3. I² statistics and meta-regression explored heterogeneity. *p*-values < 0.05 were considered significant.

**Results:**

Seven studies comprising 479 patients with 493 high-risk lesions undergoing CE-MRI were included. The average breast cancer prevalence was 17% (88/493). Pooled sensitivity was 91.3% (95% CI: 82.8–95.8%) and pooled specificity was 68.8% (95% CI: 50.3–82.8%). Only 6/493 malignancies were missed by CE-MRI; all were small low-grade ductal carcinoma in situ (DCIS). Fagan nomograms indicated that CE-MRI could rule out malignancy in lesions with pretest probabilities up to 13.1%.

**Conclusions:**

CE-MRI in assessing high-risk lesions may help identify patients who can safely avoid surgery, potentially reducing morbidity, anxiety, and healthcare resource use. Malignancy can be reliably ruled out in lesions with pretest probabilities ≤ 13.1%, although prospective studies are suggested for confirmation.

**Key Points:**

***Question*** *Can contrast-enhanced breast MRI help to rule out malignancy in patients with high-risk lesions at imaging-guided biopsy, thereby supporting more tailored decisions and potentially reducing unnecessary surgical excisions?*

***Findings*** *Contrast-enhanced breast MRI may reduce unnecessary surgical or vacuum excisions in high-risk (B3) lesions. Missed cancers were limited to small low-grade DCIS.*

***Clinical relevance*** *Contrast-enhanced breast MRI may support the identification of patients with high-risk lesions who could potentially avoid surgery. This non-invasive approach has the potential to reduce overtreatment, healthcare costs, and patient anxiety, while maintaining a high negative predictive value.*

**Graphical Abstract:**

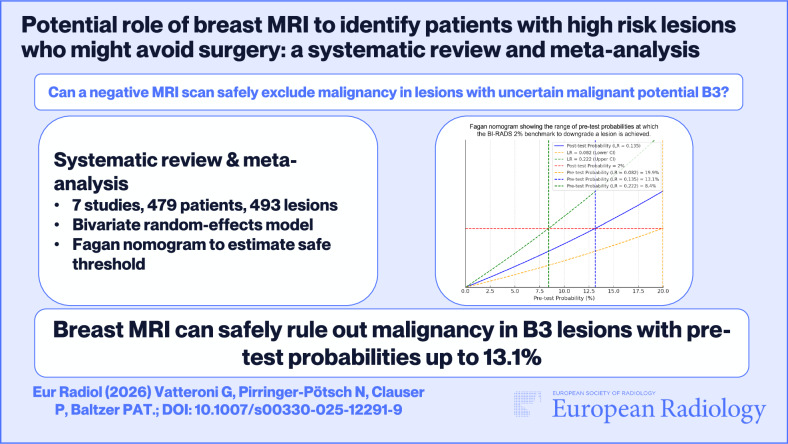

## Introduction

Breast imaging is essential for detecting breast cancer, but it also identifies a range of findings beyond malignant lesions, including benign and indeterminate lesions. The latter, also known as “B3 lesions” according to European and UK guidelines [1–3], are a heterogeneous group that poses unique diagnostic and management challenges due to the inability to exclude malignancy, often leading to unnecessary surgeries. In the US literature, the umbrella term “high-risk lesions” is typically used instead of “B3.” To ensure clarity across audiences, we use high-risk (B3) lesions throughout this manuscript. Although most of these high-risk lesions diagnosed at initial biopsy are benign, up to one-third may harbor malignancy or carry an increased risk of cancer [4, 5].

Accurate risk stratification of high-risk lesions is therefore crucial for identifying low-risk cases and minimizing invasive procedures. Contrast-enhanced magnetic resonance imaging (CE-MRI) has demonstrated high sensitivity and specificity, and a strong negative predictive value for breast lesion characterization, potentially reducing unnecessary follow-ups and biopsies as false-negative rates consistently stay below the BI-RADS 3 threshold of 2%. This also holds true in the more challenging subgroup of lesions presenting with mammographic calcifications, in which the rate of invasive cancers (1.6%) also falls under this threshold if MRI is negative [6–9].

For such reason, it would be desirable to be able to stratify the risk of high-risk lesions and downgrade those at low risk of harboring cancer, thereby avoiding unnecessary invasive procedures. CE-MRI has demonstrated high sensitivity and specificity in breast lesion characterization, avoiding unnecessary follow-up or biopsies in up to 80% due to its high negative predictive value [6–9]. However, its role in excluding malignancy in high-risk lesions remains uncertain due to limited and conflicting evidence from small and heterogeneous studies.

This systematic review and meta-analysis aimed to investigate the value of CE-MRI to rule out malignancy in high-risk lesions, with the aim of avoiding invasive removal of lesions at low risk of harboring cancer. Because high-risk subtypes differ primarily in baseline upgrade rates rather than MRI test performance, we frame MRI utility using Bayes’ theorem/Fagan nomograms: pretest probability (subtype-specific prevalence) combined with pooled negative likelihood ratios (LR−) yields post-test probability, which we compare to the BI-RADS 3 threshold (2%). This provides a transparent, clinically usable rule for considering surveillance versus excision.

## Methods

### Study design and eligibility criteria for study selection

This is a systematic review and meta-analysis of published research data on the added value of CE-MRI to rule out malignancy in patients with high-risk lesions. The research strategy was defined before the start of the study, and the analysis was performed in adherence with the Preferred Reporting Items for Systematic Reviews and Meta-Analyses (PRISMA) [10]. To be eligible, each article had to provide at least raw data from which to extract true-positive (TP), false-positive (FP), true-negative (TN), and false-negative (FN) findings to construct a 2 × 2 table. Histopathological examination or imaging follow-up of at least 12 months was defined as the required reference standard. Studies focusing on borderline lesions not dealing with CE-MRI and without a comparison with histology were considered not representative of the research question and excluded. Studies with incomplete data, meta-analyses and reviews, comments, articles not in English, and other non-related studies were also not included in the analysis. Also, studies were excluded if they reported overlapping patient populations.

### Search strategy

A computerized query and systematic review of articles in PubMed was performed by a reader (G.V., with more than 6 years of experience in breast imaging). All articles listed online up to August 21, 2024, with no lower time-point limit, were considered. The search term “breast MRI borderline b3 lesion” was used. For PubMed, the search string read (breast mri) AND ((borderline) OR (b3)) AND (lesion) : (“breast”[MeSH Terms] OR “breast”[All Fields] OR “breasts”[All Fields] OR “breast s”[All Fields]) AND (“magnetic resonance imaging”[MeSH Terms] OR (“magnetic”[All Fields] AND “resonance”[All Fields] AND “imaging”[All Fields]) OR “magnetic resonance imaging”[All Fields] OR “mri”[All Fields]) AND (“borderline”[All Fields] OR “borderlines”[All Fields] OR “b3”[All Fields]) AND (“lesion”[All Fields] OR “lesion s”[All Fields] OR “lesional”[All Fields] OR “lesions”[All Fields]).

Research articles cited in the paper by Linda et al [11], the first article that addressed the research question, were scanned on Google Scholar for additional eligible studies (“forward snowballing”).

All the titles and abstracts of the search results were then reviewed for eligibility and exported to an Excel spreadsheet. Full texts of eligible studies were retrieved and reviewed with a second reader (P.A.T.B., with 22 years of experience in breast imaging). If discrepancies occurred, they were resolved by consensus.

### Data collection and risk of bias

Two independent readers (G.V., P.A.T.B.) selected eligible studies and extracted the data in a predefined Excel spreadsheet, assessed risk of bias and applicability concerns according to Quality Assessment of Diagnostic Accuracy Studies (QUADAS)–2 [12].

The extracted data included: author, year, journal, paper title, setting, design (prospective and retrospective), indication for MRI imaging, reference standard, number of patients, number of high-risk lesions, number of malignant lesions, MRI field strength, MRI Vendor, coil, contrast agent, contrast dose, MRI sequences, number of readers and reading modality.

Extraction of imaging results (true-positive, false-positive, true-negative, and false-negative) was performed. “Positive” was defined as malignant. If the study involved more than one reader, the raw data results average was calculated and reported.

### Statistical analysis

Analyses were performed by using commercial (STATA/SE 15, StataCorp, MIDAS plugin) and open-source (OpenMeta Analyst 0.1, https://www.brown.edu/public-health/cesh/resources/software) software.

A bivariate random-effects model of combined sensitivity and specificity was used to calculate pooled effect sizes. A summary receiver operating characteristic (sROC) curve was calculated.

Sources of heterogeneity were investigated by using a random-effects meta-regression, and the study design, B3 histology, and standard of reference parameters on the diagnostic performance metrics of sensitivity, specificity, and diagnostic odds ratio were investigated. The inconsistency I^2^ was calculated and classified as low (≤ 25%), medium (≤ 50%), or high (≤ 75%).

A Fagan nomogram was used to provide post-test probabilities based on variable pretest probabilities for clinical decision-making. *p* < 0.05 was considered statistically significant.

## Results

**Fig. 1:**
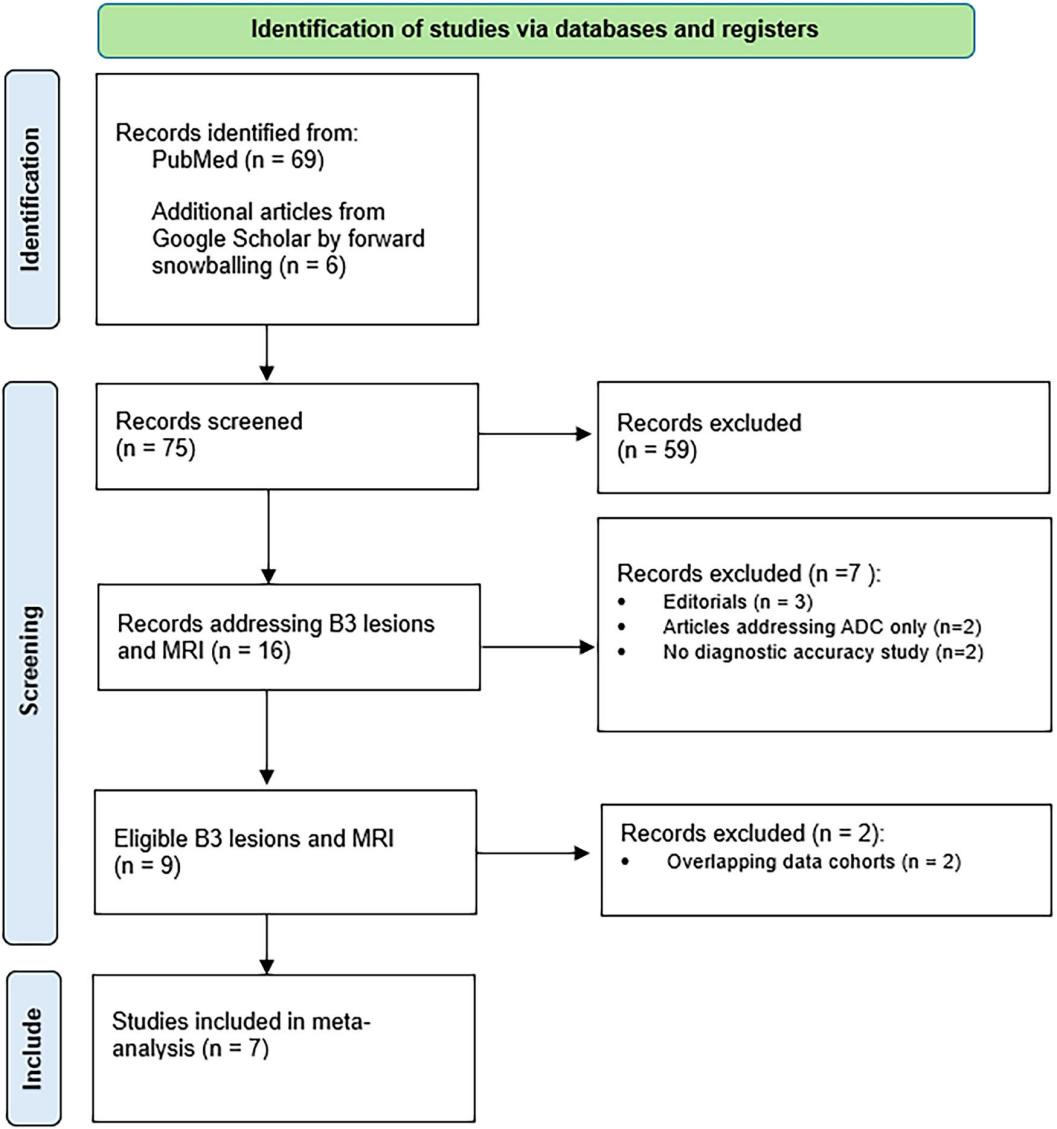
Preferred reporting items for systematic reviews and meta-analyses 2020 flowchart showing the systematic search results and the selection process toward the included studies

The study selection process is given in the PRISMA flowchart [10] (Fig. 1).

The data synthesis included seven eligible studies investigating 479 patients with 493 high-risk lesions undergoing breast MRI. The studies by Linda published in 2008 and 2012 [11, 13] were excluded since the data populations overlapped with the included study by Londero et al [14].

The cancer prevalence was 17.8% (88/493), ranging from 10.6% [15] and 52.9% [16]. The study design was retrospective in all studies but two studies [16, 17]. Patient recruitment was consecutive in three studies [14, 16, 17], while no information was available in the rest of the studies [15, 18–20]. Extracted data are summarized in Table 1.

**Table 1:** Key parameters extracted from the eligible studies

Author and year	Setting	B3 histology	Design	Reference standard	No. of lesions/no. of patients	Prevalence of malignancy (%)	TP	FP	TN	FN
Pediconi 2010	US only	Mixed	P	HE	32/32	8/32 (25.0%)	7	2	22	1
Londero 2012	Conventional imaging	Mixed	R	HE + FU	227/220	30/227 (13.2%)	28	127	70	2
Petrovecki 2016	Conventional imaging	Mixed	R	HE + FU	54/54	12/54 (22.2%)	12	10	32	0
Tsuchiya 2017	Conventional imaging	ADH	P	HE	17/17	9/17 (52.9%)	9	1	7	0
Amitai 2018	Conventional imaging	ADH	R	HE + FU	48/48	7/48 (14.5%)	6	7	34	1
Hammersley 2018	Conventional imaging	Mixed	R	HE + FU	47/42	5/47 (10.6%)	4	21	21	1
Bertani 2020	Conventional imaging	ADH	R	HE	68/66	17/68 (25.0%)	16	20	31	1

*No.* number, *NA* not available, *TP* true positive, *FP* false positives, *TN* true negatives, *FN* false negatives, *ADH* atypical ductal hyperplasia, *R* retrospective, *P* prospective, *HE* histological examination, *FU* follow-up of at least 24 months

All the studies involved 2 readers blinded to the histopathological results: independent reading was performed in three studies [14–16], while in three other studies [17, 19, 20] readings were performed in consensus. The study by Petrovecki et al [18] did not provide any information about how the readings were performed. Among the studies where the readings were performed independently, the kappa agreement was assessed only in the study by Hammersley et al [15], which was moderate. Londero et al [14] and Tsuchiya et al [16] reported that discordant cases were solved by consensus.

All the studies used a dedicated breast coil and examined the patients in the prone position, except for two studies that did not provide any information regarding this point [15, 18].

All the studies, except the one by Petrovecki et al [18], specified the MRI acquisition protocol. Study-specific characteristics are given in Table 2.

**Table 2:** Study-specific characteristics

Author and year	Field strength (T)	Vendor	Coil	Contrast agent	CM dose (mmol/kg)	MRI protocol
Pediconi 2010	1.5	Siemens	Dedicated breast coil	Gadobenate dimeglumine	0.1	Axial dynamics, fatsat
Londero 2012	1.0–1.5	Siemens	7 channel 2 channel	Gadopentetate dimeglumine Gadobenate dimeglumine	0.1	Coronal dynamics, no fatsat
Petrovecki 2016	n.a.	n.a.	n.a.	n.a.	n.a.	n.a.
Tsuchiya 2017	3	Philips	16 channel	Gadobutrol	0.1	Axial dynamics, fatsat
Amitai 2018	1.5–3	GE Siemens	Dedicated Breast coil	Gadolinium-based	0.2	Axial dynamics, fatsat
Hammersley 2018	1.5–3	GE Siemens	n.a.	Gadobenate dimeglumine Gadodiamide	0.1	Sagittal, axial dynamics, fatsat, no subtraction
Bertani 2020	1.5–3	GE	8 channel	Gadoterate meglumine	0.1	Axial dynamics standard, fatsat

*CM* contrast medium, *fatsat* fat suppressed, *n.a.* not available

**Fig. 2:**
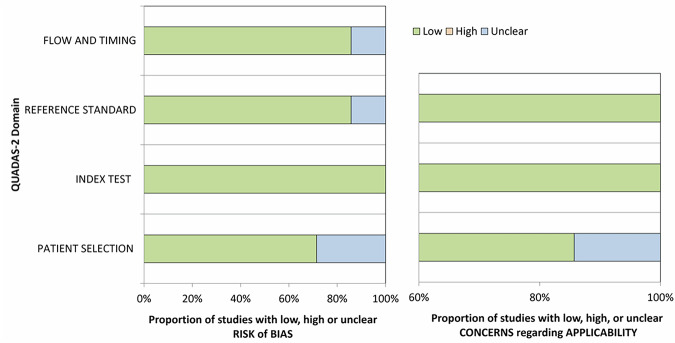
QUADAS-2 bias risk assessment

QUADAS assessment is summarized in Fig. 2. With respect to patient selection, the risk of bias was rated unclear in 2 studies [14, 18] while in the other studies, a low risk of bias was present. Regarding the index test, a low risk of bias was estimated. Reference standard and flow and timing were unclear in one study [18]. There were no concerns regarding the applicability of the included studies to the research questions.

### Diagnostic performance data synthesis

**Fig. 3:**
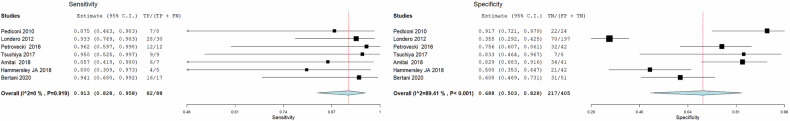
Forest plot of sensitivity and specificity data synthesis using a random-effects model

**Fig. 4:**
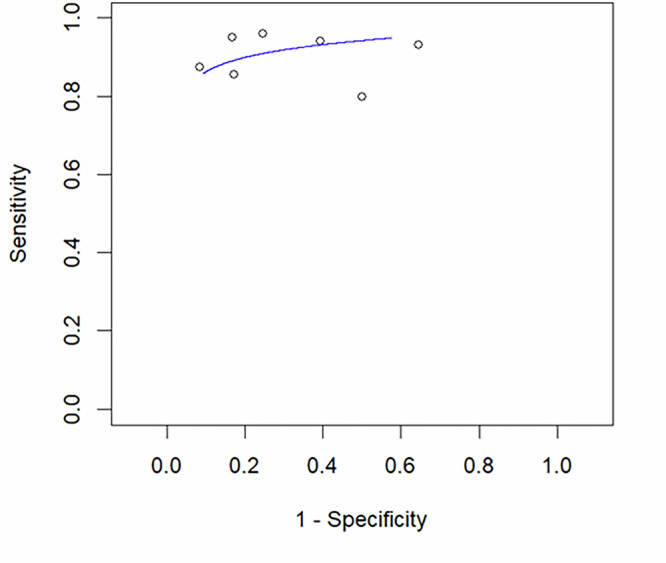
Summary ROC curve derived from bivariate modeling of sensitivity and specificity from individual studies

Considering all the lesions, pooled sensitivity was 91.3% (95% CI: 82.8–95.8%) according to random effect model, ranging from 80.0% [15] to 96.2% [18]. Pooled specificity was 68.8% (95% CI: 50.3–82.8%), calculated by using a random effect model. Specificity ranged from 35.5% [14] to 91.7% [17]. Random effect model results of diagnostic performance metrics and the sROC curve derived from bivariate data modeling are shown in Figs. 3 and 4, respectively.

A sub-analysis was conducted regarding study design. In the subgroup of prospective studies [16, 17] according to random effect model pooled sensitivity was 90.9% (95% CI: 64.8–98.2%) ranging from 87.5% [17] to 95.0% [16], while pooled specificity was 88.9% (95% CI: 72.4–96.1%) ranging from 83.3% [16] to 91.7% [17]. In the retrospective studies group [14, 15, 18–20] pooled sensitivity was 91.3% (95% CI: 82.8–95.8%) comprised between 80.0% [15] and 96.2% [18], while pooled specificity was 68.8% (95% CI: 50.3–82.8%) comprised between 35.5% [14] and 82.9% [19] (Supplementary Fig. 1).

We conducted a sub-analysis regarding high-risk histology. In the subgroup comprising studies with mixed histology, pooled sensitivity was 91.0% (95% CI: 79.1–96.4%) and pooled specificity was 63.9% (95% CI: 38.3–83.5%). In studies addressing only ADH [16, 19, 20], pooled sensitivity was 92.0% (95% CI: 75.7–97.7%) and pooled specificity was 74.6% (95% CI: 54.2–87.9%) (Supplementary Fig. 2).

**Fig. 5:**
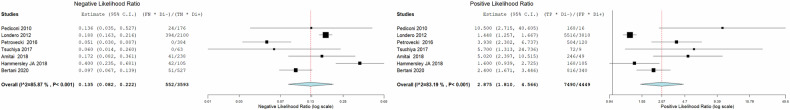
Forest plot of negative likelihood ratio and positive likelihood ratio values based on random-effects data synthesis

The pooled negative likelihood ratio was 0.135 (95% CI: 0.082–0.222) while the pooled positive likelihood ratio was 2.875 (95% CI: 1.810–4.566) (Fig. 5).

### Sources of heterogeneity

Between the included studies, there was not a statistically significant heterogeneity for sensitivity (I^2^ = 0%, *p* = 0.919), while a significant heterogeneity was demonstrated regarding specificity (I^2^ = 89.41%, *p* < 0.001).

Investigating the possible source of heterogeneity, neither prevalence, study design, high-risk type and standard of reference influenced diagnostic parameters upon meta-regression (*p* > 0.05, each).

### Details of false-negative findings among invasive cancers and all malignant lesions

All the studies provided the number and histology of missed cancers. There were no invasive cancers missed by MRI in any study, resulting in an NPV for invasive cancer of 100%. All the false-negative results were small low-grade DCIS: one study missed 2 non-invasive cancers [14], four studies missed only one intraductal carcinoma [15, 17, 19, 20] while two studies did not miss any cancer [16, 18].

### Pretest probability of MRI for ruling out malignancy (invasive cancer and DCIS)

**Fig. 6:**
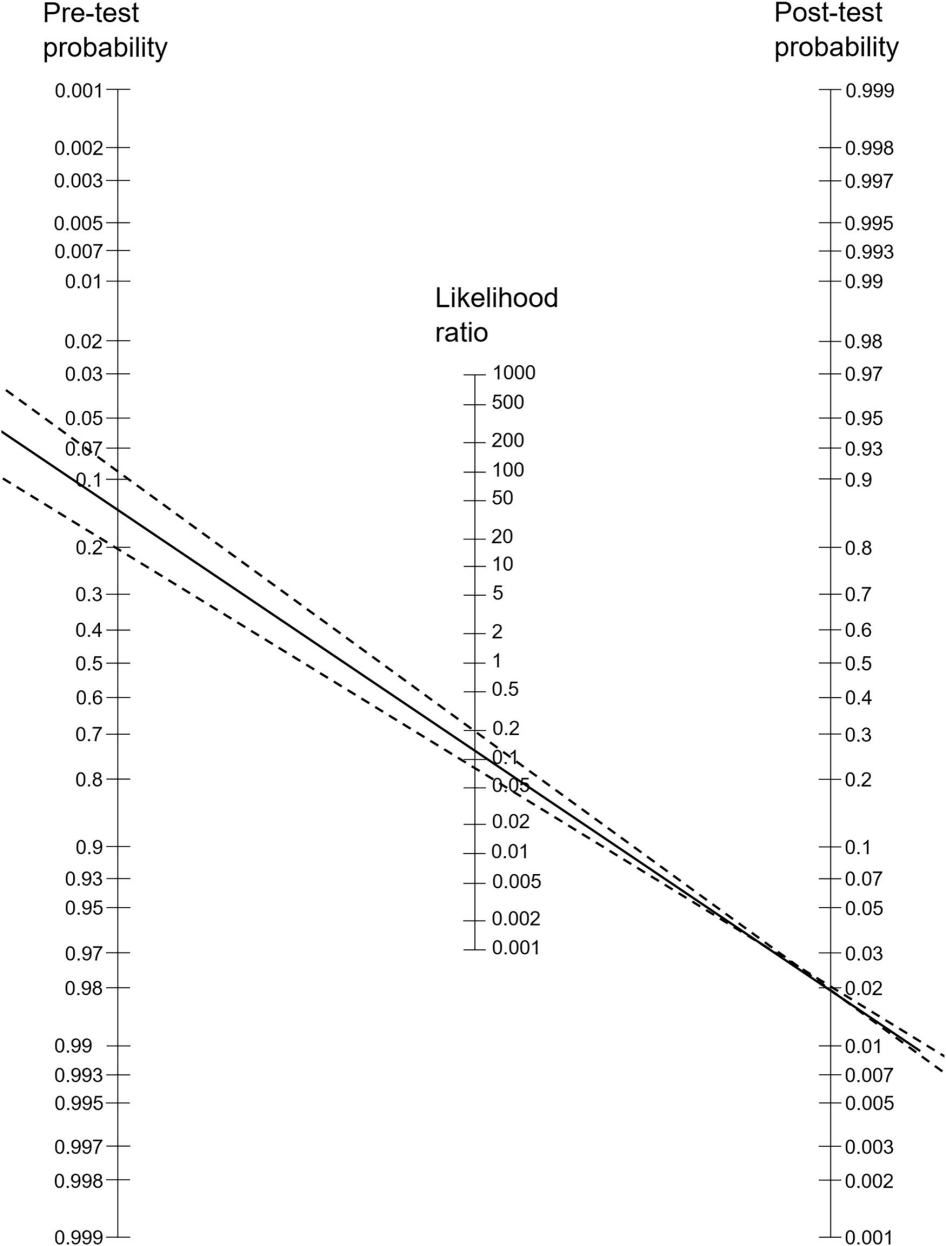
A Fagan nomogram demonstrating the study results for immediate application. The solid line represents the pooled LR- summary estimate, dashed lines the 95% CI. The post-test probability was fixed at the 2% benchmark for BI-RADS 3 (right axis). Thereby, the reader can see in which pretest probability range (left axis) a rule out of malignancy is possible. The practical clinical pathway would be as follows: high-risk lesion on image-guided biopsy → CE-MRI performed → look up subtype-specific pretest probability from the literature referenced in text→ if this is within the range indicated on the left → consider imaging surveillance or return to follow-up after considering the individual situation and patient wishes in shared decision-making; if > 2% → proceed to excision as local standard

To estimate the ability of a negative MRI to rule out malignancy in high-risk lesions, we applied the pooled negative likelihood ratios to a Fagan nomogram (Fig. 6). Up to a pretest probability of 13.1% (range 8.4–19.9%), a negative MRI resulted in a post-test probability of ≤ 2%, allowing to downgrade the lesion while meeting BI-RADS 3 benchmarks.

## Discussion

This systematic review and meta-analysis show that CE-MRI could accurately rule out invasive cancer in high-risk lesions, thereby demonstrating the potential to avoid unnecessary surgery. Only non-invasive cancers were missed, while a pooled estimate of 68.8% (95% CI: 50.3–82.8%) of unnecessary surgeries/vacuum-resections could have been avoided. Given the natural history of DCIS, these findings suggest that MRI could safely rule out malignancy in a majority of high-risk lesions [21]. This is highly relevant since high-risk lesions comprise a significant and rising amount of biopsy specimens, and their management is both more costly and invasive than in other breast lesions.

Recently developed European guidelines for the diagnosis, treatment and follow-up of lesions with uncertain malignant potential [22] highlight that this is a heterogeneous group comprising lesions with different malignant potential and progression risk, ranging from 1 to 20% for DCIS and 1 to 7% for invasive cancer. Besides, large databases have shown an increasing incidence of high-risk lesions in the last decades: from 5–9% (1999–2006) [23], 17.0% (2009–2011) [24] to 19.1% (2007–2017) [25].

In practical terms, pretest probabilities can be estimated from the published upgrade rates of high-risk subtypes. This allows direct application of our results, pointing out only ADH and lobular neoplasia as lesion types that typically exceed the BI-RADS 3 category 2% post-test probability for breast cancer. Large databases [5, 23–36] show that a negative CE-MRI could exclude invasive cancer in practically all high-risk lesions [22]. CE-MRI can largely rule out any malignancy in Radial Scar/complex sclerosing lesions [31, 32], Flat Epithelial Atypia [29, 30] and intraductal papilloma [26, 33], while it may not have the potential role to exclude any cancer in ADH [35] and in lobular neoplasia (particularly in atypical lobular hyperplasia) [27, 28] since the total upgrade rate of these lesions is respectively up to 50% and 22%. These last two entities are generally considered surgical, not only for the upgrade rate at subsequent surgery but also for the increased lifetime risk of developing breast cancer up to 5 times in ADH and 10 times in lobular neoplasia [22].

Notably, in the analyzed studies [14, 15, 17, 19, 20], cancers not diagnosed by CE-MRI were all small low-grade DCIS. CE-MRI is, in fact, more sensitive in detecting clinically significant and biologically aggressive carcinomas [37], meaning that we could safely apply a “wait and see” approach in a lesion that does not show a suspicious enhancement on CE-MRI, without changing patients’ prognosis but in the worst hypothesis, leading to a delayed diagnosis in a biologically not significant cancer.

Our findings have high clinical relevance as they could be leveraged to avoid unnecessary further interventions, including open surgery under general anesthesia. The latter is in fact associated with a risk of post-operative major complications [38] as well as psychological impact on women. Our meta-analysis shows consistently high NPV and low LR– for MRI, supporting its value in potentially avoiding surgery in a major fraction of high-risk cases. Although this reduces morbidity, costs, and psychological burden, some patients may prefer surgical certainty; hence, we believe shared decision-making is crucial for the translation of the reported findings into clinical practice. Given that the prevalence of malignancy in MRI-negative high-risk lesions is < 2% and no invasive cancers were identified in our meta-analysis, structured imaging follow-up (e.g., at 1–2 years, practically a return to screening) appears to be a reasonable and safe alternative to surgical excision.

Although formal cost-effectiveness analyses were beyond the scope of this study, reported costs of surgery depend on the European country and healthcare system, ranging from several thousand Euros for Breast Conserving surgery, but also for VABB resection, while an MRI cost is typically below 500 Euros [39].

In the systematically reviewed literature, we noted some research gaps.

First of all, a significant limitation in the reviewed studies is the absence of standardized imaging diagnostic criteria. The performance of diagnostic tools and decision-making could, in fact, benefit greatly from a structured clinical decision rule that systematically combines diagnostic criteria. Future studies should prioritize the development and validation of such frameworks to improve diagnostic accuracy and consistency.

Second, many studies did not provide detailed information on the radiological characteristics of high-risk lesion subtypes or specify the imaging modality through which these lesions were initially detected (Table 3). For such reasons, investigating these aspects could enhance our understanding of imaging patterns associated with high-risk lesions, enabling improved risk stratification and tailored management approaches. Third, in the literature, the methodologies used for patient selection were often underreported or unclear in the included studies. For example, it remains uncertain whether selection was biased toward patients with imaging findings suspicious for malignancy. Research exploring how patient populations are defined and selected could address these biases, ensuring more representative and generalizable results are warranted. Moreover, the variability in upgrade rates (transition from high-risk to malignant diagnoses) may be influenced by both the surgeon’s expertise and the biopsy technique used. Despite its clinical significance, no studies have systematically explored how these factors affect outcomes. Investigating this gap could enhance procedural guidelines and improve diagnostic consistency.

**Table 3:** Extracted data on radiological appearance on MRI

Author and year	Absent enhancement	Mass	NME	Foci
Pediconi 2010	0	23	9	0
Londero 2012	72	125	21	9
Petrovecki 2016	n.a.	n.a.	n.a.	n.a.
Tsuchiya 2017	7	0	10	0
Amitai 2018	35	3	10	0
Hammersley 2018	n.a.	n.a.	n.a.	n.a.
Bertani 2020	32	5	31	0

*NME* non-mass enhancement, *n.a.* not available

From our literature research, it is also evident that data on long-term outcomes of patients with high-risk lesions are sparse, and there is a need for studies that investigate optimal follow-up intervals and frequencies to better understand the natural history of these lesions and improve long-term patient management to establish evidence-based surveillance protocols.

Our study had some limitations. First, there was high heterogeneity between the included studies, which we could not fully explain due to the limited data that represent the main research gap. Likely contributors are differences in cancer prevalence, imaging protocols, and interpretive variability. However, since the primary clinical question relates to the safety of downgrading (sensitivity/NPV), the impact of this heterogeneity is limited; specificity is more relevant for evaluating cost-effectiveness and potential reductions in surgery. Furthermore, a lack of standardized MRI protocols and interpretive criteria across studies was evident. While this heterogeneity is a limitation, it also reflects real-world practice and emphasizes the need for future research using uniform diagnostic thresholds. If, for instance, the absence of enhancement was a suitable rule-out criterion, alternative techniques such as contrast-enhanced mammography could be applied in practice. Although pretest probabilities differ among high-risk subtypes, there is no evidence that MRI diagnostic performance itself varies by histology. Likelihood ratios can therefore be validly applied across subgroups to derive post-test probabilities. Given the variable performance of MRI in lesions depending on whether there are mammographic calcifications or not, it would be most valuable to distinguish between high-risk with or without mammographic calcifications. Such data are not available, though. An expanded search using histology-specific keywords (ADH, ALH/LCIS, FEA, intraductal papilloma, radial scar/CSL) and calcification terms, in addition to high-risk (B3) lesions, did not identify additional CE-MRI index-test primary studies with extractable 2 × 2 data. Most available studies were retrospective, which reflects the clinical reality that high-risk lesions are diagnosed on biopsy, while MRI is usually performed for other indications. Although this limits control over patient selection, it underscores the current evidence gap and highlights the need for prospective studies specifically designed to assess MRI in high-risk management.

In addition, our results are both promising and plausible as they are in line with prior meta-analyses [6, 9], which demonstrated that MRI can reliably exclude prognostically relevant malignancy in diverse settings. It should be noted that high-risk lesions have a theoretical malignant potential over extended timeframes. Currently, no evidence regarding a progression to invasive cancer after MRI-based downgrading has been reported. Still, prospective trials with longer follow-up are required to better define follow-up intervals after a negative MRI, analogous to screening practice.

## Summary and conclusion

In conclusion, this systematic review and meta-analysis highlight the potential value of CE-MRI in the assessment of high-risk lesions. This approach shows very promising results and high clinical relevance, potentially identifying patients who might avoid surgery, saving costs and time, as well as reducing patient anxiety and morbidity. Given that most existing studies are retrospective, our findings provide a necessary foundation for future prospective research in this field to investigate the impact of false-negative findings on clinical management and patient outcomes.

## Supplementary information

Supplementary information

## Abbreviations

CE-MRI: Contrast-enhanced MRI
DCIS: Ductal carcinoma in situ
FN: False negatives
FP: False positives
LR−: Pooled negative likelihood ratios
PRISMA: Preferred Reporting Items for Systematic Reviews and Meta-Analyses
sROC: Summary receiver operating characteristics
TN: True negatives
TP: True positives
