# Supplementary material for: Potential role of breast MRI to identify patients with high-risk lesions who might avoid surgery: a systematic review and meta-analysis
Source: Eur Radiol. 2026 Jan 17;36(6):4434–44. doi: 10.1007/s00330-025-12291-9 (PMC13212820; doi:10.1007/s00330-025-12291-9)

Potential role of Breast MRI to identify patients with high-risk lesions who might avoid surgery: a systematic review and metanalysis

ELECTRONIC SUPPLEMENTARY MATERIAL

Supplementary Figure 1

Forest plot of sensitivity and specificity data synthesis using a random effects model in a sub-analysis regarding study design (prospective P against retrospective R).

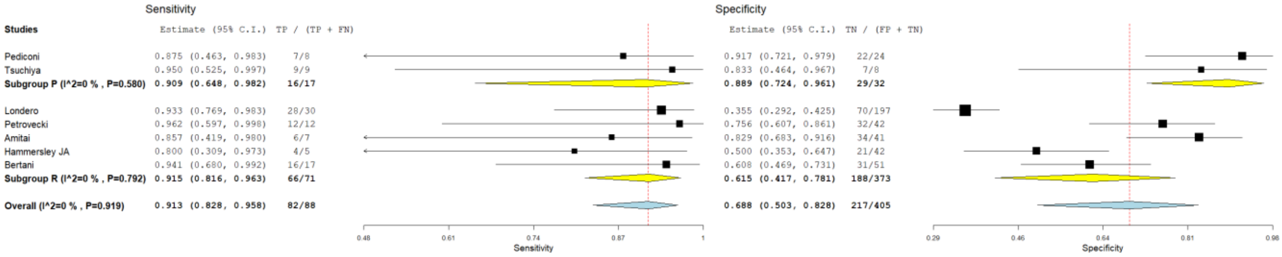

Supplementary Figure 2

Forest plot of sensitivity and specificity data synthesis using a random effects model in a sub-analysis regarding B3 histology (mixed vs ADH)

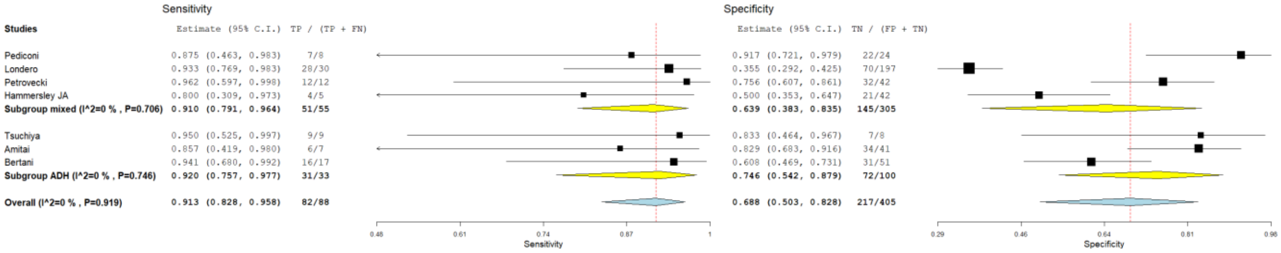

Supplement: Supplementary file 1 — Supplementary information [file 330_2025_12291_MOESM1_ESM.pdf]
